# Inhibition of TBC1D5 activates Rab7a and can enhance the function of the retromer cargo-selective complex

**DOI:** 10.1242/jcs.217398

**Published:** 2018-06-21

**Authors:** Matthew N. J. Seaman, Aamir S. Mukadam, Sophia Y. Breusegem

**Affiliations:** Department of Clinical Biochemistry, Cambridge Institute for Medical Research, University of Cambridge, Addenbrookes Biomedical Campus, Cambridge, CB2 0XY, UK

**Keywords:** Retromer, TBC1D5, Rab7, Activation, Endosome, Sorting

## Abstract

The retromer complex is a vital component of the endosomal protein sorting machinery necessary for sorting into both the endosome-to-Golgi retrieval pathway and also the endosome-to-cell-surface recycling pathway. Retromer mediates cargo selection through a trimeric complex comprising VPS35, VPS29 and VPS26, which is recruited to endosomes by binding to Rab7a and Snx3. Retromer function is linked to two distinct neurodegenerative diseases, Parkinson's disease and Alzheimer's disease and modulating retromer function has been proposed as an avenue to explore for a putative therapy in these conditions. We hypothesised that activating Rab7a to promote the recruitment of retromer to endosomes could positively modulate its activity. Here, we show that inhibition of the GTPase activating protein TBC1D5 can enhance Rab7a activation and lead to a gain of function for retromer.

## Abstract (precis)

**Highlighted Article:** Enhancement of retromer complex function through inhibition of TBC1D5, a Rab GTPase-activating protein for Rab7a, leads to a gain of function for retromer-mediated endosomal sorting.

## INTRODUCTION

The retromer complex is a key player in endosomal protein sorting and is conserved across all eukaryotes. Retromer comprises two functional units that, in higher eukaryotes such as mammals, associate loosely to mediate the sorting and transport of proteins from endosomes to either the Golgi or the cell surface (Seaman, 2012; Burd and Cullen, 2014). A stable trimeric complex of VPS35, VPS29 and VPS26 is important in selecting membrane proteins (cargo) and is often referred to as the cargo-selective complex (CSC). Formation of tubular carriers is mediated by a dimer of Bin/amphiphysin/Rvs domain-containing sorting nexin (SNX-BAR) proteins containing either Snx1 or Snx2 bound to Snx5 or Snx6 (van Weering et al., 2012). The SNX-BAR proteins have also been reported to interact with some cargo proteins, notably the cation-independent mannose 6-phosphate receptor (CIMPR) (Kvainickas et al., 2017; Simonetti et al., 2017).

The SNX-BAR dimer can associate with endosomes by binding to phosphatidyl inositol 3-phosphate (PtdIns3P) but the retromer cargo-selective complex (CSC) requires Rab7a and Snx3 for recruitment to endosomes (Cozier et al., 2002; Rojas et al., 2008; Seaman et al., 2009; Harterink et al., 2011; Vardarajan et al., 2012; Harrison et al., 2014). In addition to associating with Rab7a and Snx3, the CSC also functions as a hub to recruit additional endosomal protein sorting machinery (Harbour et al., 2010; Hesketh et al., 2014). The Wiskott-Aldrich syndrome protein and Scar homolog (WASH) complex mediates formation of filamentous (F)-actin and requires interaction between VPS35 and the WASH complex protein Fam21 (also known as WASHC2A) for its membrane association (Harbour et al., 2012; Jia et al., 2012; Helfer et al., 2013). VPS9-ankyrin-repeat protein (VARP, also known as ANKRD27) serves as a guanine nucleotide exchange factor (GEF) for Rab21 and an effector for Rab32 and Rab38, and binds to VPS29, targeting it to endosomes (Hesketh et al., 2014). In addition, the Rab GTPase-activating protein (GAP) TBC1D5 also requires the retromer CSC for its membrane association, and interestingly, binds to VPS29 via the same hydrophobic surface patch as VARP does (Harbour et al., 2010; Hesketh et al., 2014). Overexpression of TBC1D5 causes the retromer CSC to be displaced from the membrane in a similar manner to the expression of the GDP-locked (and therefore inactive) T22N mutant of Rab7a and thus it has been suggested that TBC1D5 could function as a GAP for Rab7a where the retromer CSC is localised (Seaman et al., 2009).

The functioning of the retromer complex has been linked to two distinct neurological diseases, Parkinson's disease (PD) and Alzheimer's disease (AD) (Small and Petsko, 2015; McMillan et al., 2017). A mutation in VPS35 (D620N) that causes an inherited form of PD results in reduced association of the WASH complex with the retromer CSC and therefore less WASH complex is recruited to the endosome (Zavodszky et al., 2014). This is because the D620N mutation reduces the binding affinity of VPS35 for the WASH complex protein Fam21 (McGough et al., 2014). The AD-linked mutation in VPS35 destabilises the retromer CSC by impairing the binding of VPS35 to VPS29 (Rovelet-LeCrux et al., 2015). It has been reported that levels of the retromer CSC are reduced in the brains of AD patients and knockdown of VPS35 results in increased processing of amyloid precursor protein (APP) to the pro-aggregatory neurotoxic Aβ peptide (Small et al., 2005; Muhammad et al., 2008). Variants of the Snx3 and Rab7a genes required for the recruitment of the retromer CSC are linked to late-onset AD and hence retromer function has been of interest to researchers investigating the underlying causes of AD (Vardarajan et al., 2012; Small, 2008). Indeed it has been shown that a pharmacological chaperone that can bind to VPS35 at the VPS35-VPS29 interface enhances the stability of the retromer CSC increasing levels in neuronal cells and reducing the processing of APP to Aβ (Mecozzi et al., 2014).

We wondered whether retromer function could be enhanced if recruitment of the retromer CSC to membranes was stimulated. Rab7a is required for recruitment of the retromer CSC and as a GTPase, its activity is regulated by specific GEFs and GAPs making it more amenable to modulation of its activity than Snx3 (Pfeffer, 2017). A good candidate for a Rab7a GAP is the TBC1D5 protein (Jia et al., 2016), and studies in nematode have supported a role for TBC1D5 in regulating the worm equivalent of Rab7a (Mukhopadhyay et al., 2007). Therefore, we investigated whether loss of TBC1D5 could boost the levels of retromer associated with endosomes and thereby enhance retromer function. Here, we report that loss of TBC1D5 does elevate the levels of GTP-bound Rab7a, increasing the association of the retromer CSC with endosomes. This leads to an enhanced interaction of retromer with accessory factors such as the WASH complex and can rescue the effect of the PD-causing VPS35 D620N mutant, generating a gain-of-function phenotype with respect to processing of APP to Aβ.

## RESULTS

### Loss of TBC1D5 function enhances retromer CSC recruitment

**Fig. 1.**
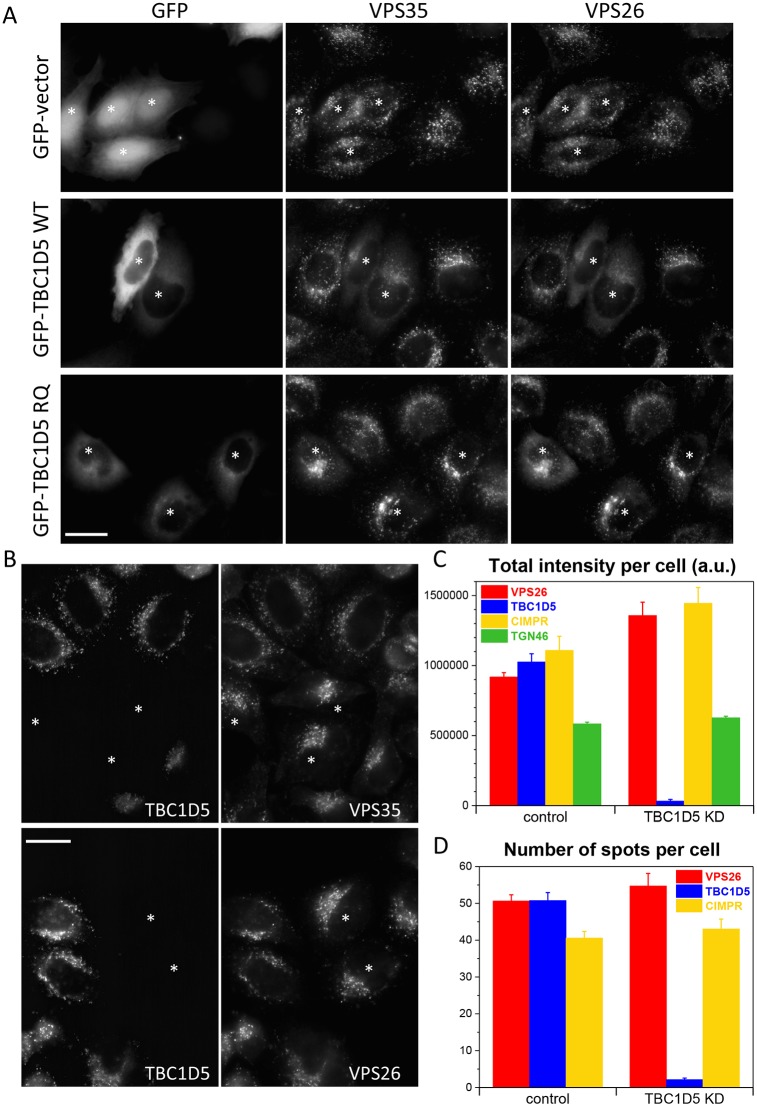
**Loss of TBC1D5 expression enhances endosomal levels of the retromer CSC.** (A) HeLa cells were transiently transfected with empty GFP vector, GFP-TBC1D5 wild type (WT) or GFP-TBC1D5 R169A/Q204A (RQ) mutant. After fixation, the cells were stained with antibodies against VPS35 and VPS26. Transfected cells are marked with an asterisk. Overexpression of the wild-type TBC1D5 can displace the retromer CSC from membranes. (B) HeLa cells were treated with siRNA to silence TBC1D5 expression. The knockdown cells were mixed with control cells and seeded onto coverslips. After fixation, cells were labelled with anti-TBC1D5 and antibodies against either VPS35 or VPS26. Loss of TBC1D5 expression (in cells marked with an asterisk) results in brighter staining of the retromer CSC proteins. (C,D) HeLa cells treated with siRNA to silence TBC1D5 expression were labelled with antibodies against VPS26, TBC1D5, CIMPR or TGN46 and then imaged using an automated microscope. Loss of TBC1D5 results in ∼40% increase in VPS26 fluorescence intensity but does not markedly increase the number of VPS26-positive spots (D). No spots were counted for TGN46 as the morphology of the TGN is not punctate but ribbon-like. *P*-values for TBC1D5 knockdown versus control: VPS26, 1.2×10^−4^; CIMPR, 0.0095; TGN46, 0.0037 for total intensity values. The *P*-values for spot numbers are: 0.08 for VPS26 and 0.23 for CIMPR. Scale bars: 20 µm.

We have previously reported that overexpression of TBC1D5 leads to reduced association of the retromer CSC with endosomes (Seaman et al., 2009). We repeated our previous observation and show that transient overexpression of wild-type GFP-tagged TBC1D5 results in reduced punctate endosomal staining of both VPS35 and VPS26 (Fig. 1A, asterisks). Previous structural studies revealed that critical residues are required for the activity of TBC proteins (Pan et al., 2006). Expression of the catalytically inactive TBC1D5 RQ mutant does not generate the same dominant-negative effect, however, suggesting that the TBC1D5 protein is exerting its effect through a Rab GTPase. As overexpression of TBC1D5 is deleterious to the endosomal localisation of the retromer CSC, we next tested whether knockdown of TBC1D5 could enhance membrane association. Control cells were mixed with TBC1D5-knockdown cells and then stained with antibodies against TBC1D5 and either VPS35 or VPS26 (Fig. 1B). Loss of TBC1D5 appears to lead to somewhat brighter staining of VPS35 and VPS26 (asterisks). To more quantitatively investigate the effect of loss of TBC1D5 on the membrane association of the retromer CSC, we used automated microscopy as in our previous study in which the role of Rab7a and Snx3 in regulating recruitment of the CSC was reported (Vardarajan et al., 2012; see also Breusegem and Seaman, 2014). This approach enables the imaging of hundreds of cells whilst avoiding unintended bias that may occur through manual imaging. Control cells and cells treated to silence TBC1D5 expression were labelled with antibodies against VPS26, TBC1D5 the CIMPR and TGN46. The cells were imaged using an automated microscope and the fluorescence intensity quantified (Fig. 1C). Loss of TBC1D5 expression increases the total intensity of VPS26 staining by ∼40% and is statistically significant (*P*-values are shown in the figure legend). The fluorescence intensity of the CIMPR also increased, but the fluorescence intensity of TGN46 was only marginally increased. A gain in total intensity could be due to increased signal per spot/endosome, or more spots. Therefore, we also plotted the average number of spots per cell (Fig. 1D). There was a modest increase in the number of VPS26 spots but the increase was not statistically significant.

**Fig. 2.**
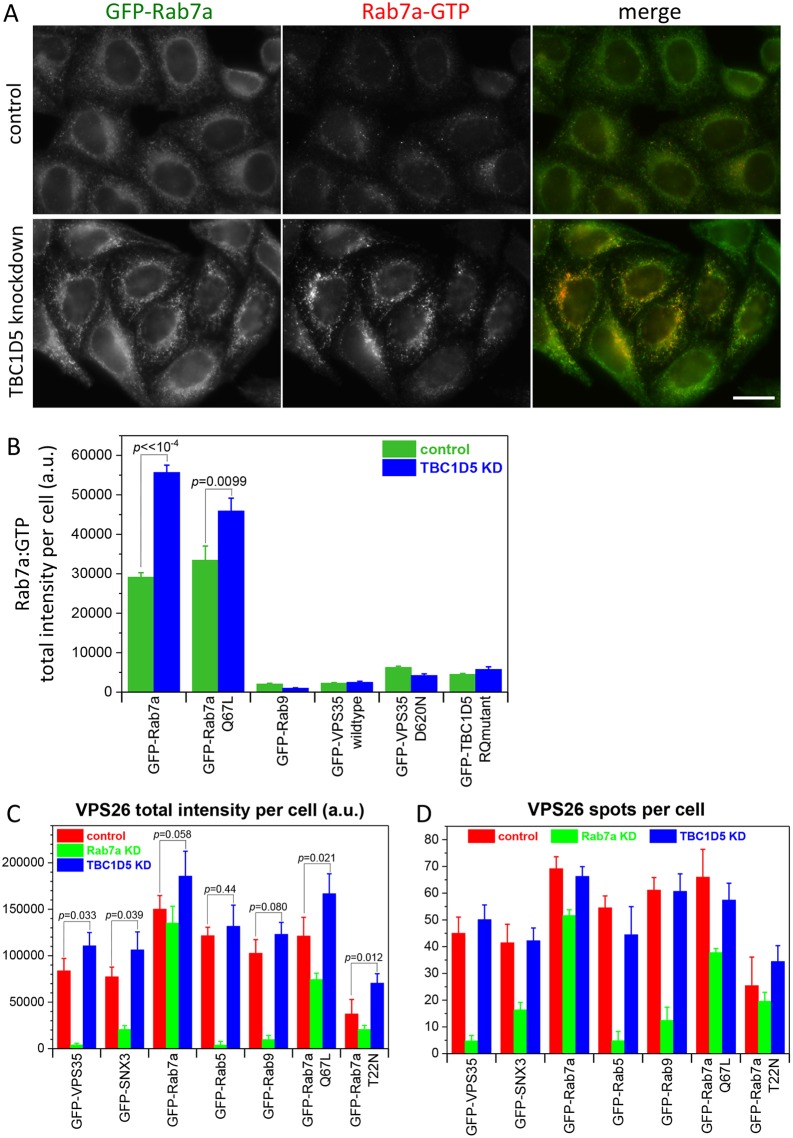
**Loss of TBC1D5 function leads to increased levels of active Rab7a.** (A) HeLa cells stably expressing GFP-tagged Rab7a-GTP were treated with siRNA to silence TBC1D5 expression. Following fixation, the cells were labelled with anti-Rab7a-GTP antibodies. Knockdown of TBC1D5 leads to increased staining of the anti-Rab7a antibody. Scale bar: 20 µm. (B) Cells stably expressing various GFP-tagged proteins were treated as in A and then imaged using an automated microscope. Only cells expressing GFP-Rab7a or GFP-Rab7a Q67L registered significant fluorescence and the knockdown of TBC1D5 results in a pronounced increase in the levels of active (GTP-bound) Rab7a. Values are mean±s.e.m. of 250 cells measured for each cell line. The *P*-values for control and TBC1D5 knockdown for cells expressing GFP-Rab7a are shown on the graph and demonstrate that the increase in fluorescence is statistically significant. (C) Cells expressing various GFP-tagged proteins were treated with siRNA to abolish either Rab7a or TBC1D5 expression. After fixation, the cells were labelled with antibodies against VPS26 and imaged using an automated microscope. Loss of Rab7a expression causes VPS26 (and the retromer CSC) to dissociate from endosomes, massively reducing the fluorescence intensity except where the knockdown of Rab7a is rescued by GFP-tagged Rab7a or Rab7a Q67L. Loss of TBC1D5 expression enhances VPS26 fluorescence even in cells where VPS26 fluorescence is lower due to the expression of a GDP-locked Rab7a T22N mutant. *P*-values are shown on the graph. (D) The number of VPS26 spots is not significantly altered after loss of TBC1D5 expression. *P*-values for all control versus TBC1D5-knockdown measurements were >0.1.

If loss of TBC1D5 function is enhancing the endosomal localisation of the retromer CSC by activating Rab7a, it should be possible to detect elevated levels of active Rab7a. Therefore, cells expressing GFP-Rab7a were treated with siRNA to ablate TBC1D5 expression. Control and knockdown cells were labelled with antibodies that specifically recognise GTP-bound Rab7a. The signal from the anti-Rab7a-GTP antibody was low in control cells (Fig. 2A, top) but markedly enhanced in cells where TBC1D5 expression had been abolished (bottom). The increase in fluorescence intensity of the Rab7a-GTP signal was quantified using automated microscopy for cells expressing a range of GFP-tagged proteins (Fig. 2B). Only where GFP-Rab7a is expressed could a signal for the anti-Rab7a-GTP antibody be detected and loss of TBC1D5 expression resulted in a significant increase in that signal (nearly two-fold for cells expressing GFP-Rab7a).

Expression of a GDP-locked and constitutively inactive Rab7a (T22N) results in reduced endosomal retromer CSC that is similar to what is observed upon overexpression of wild-type TBC1D5 (Seaman et al., 2009). Thus, we next tested whether loss of TBC1D5 function could enhance levels of retromer CSC in cells stably expressing the Rab7aT22N mutant by silencing TBC1D5 expression in a panel of seven different cell lines expressing GFP-tagged proteins. In Fig. 2C, loss of TBC1D5 expression increases the VPS26 fluorescence intensity across all the cell lines tested, but in cells expressing GFP-Rab7a, GFP-Rab5 or GFP-Rab9, the increase was not statistically significant. There was, however, a statistically significant increase in cells expressing the GDP-locked Rab7aT22N mutant, most likely due to increased activation of endogenous Rab7a that is present in the cell line. Similarly, the cells expressing the constitutively active Rab7a Q67L mutant also demonstrated an increase in endosomal VPS26 after TBC1D5 knockdown, as these cells contain endogenous Rab7a in addition to the stably expressed GFP-tagged version. Knockdown of Rab7a reduced the VPS26 fluorescence intensity, except for experiments where Rab7a was expressed as a GFP-tagged protein: as the Rab7a in this construct is of murine origin, it is resistant to the Rab7a siRNA we used. The gain in total fluorescence for VPS26 was again not due to increased spot number (see Fig. 2D).

### Enhancement of retromer recruitment increases association with accessory proteins

**Fig. 3.**
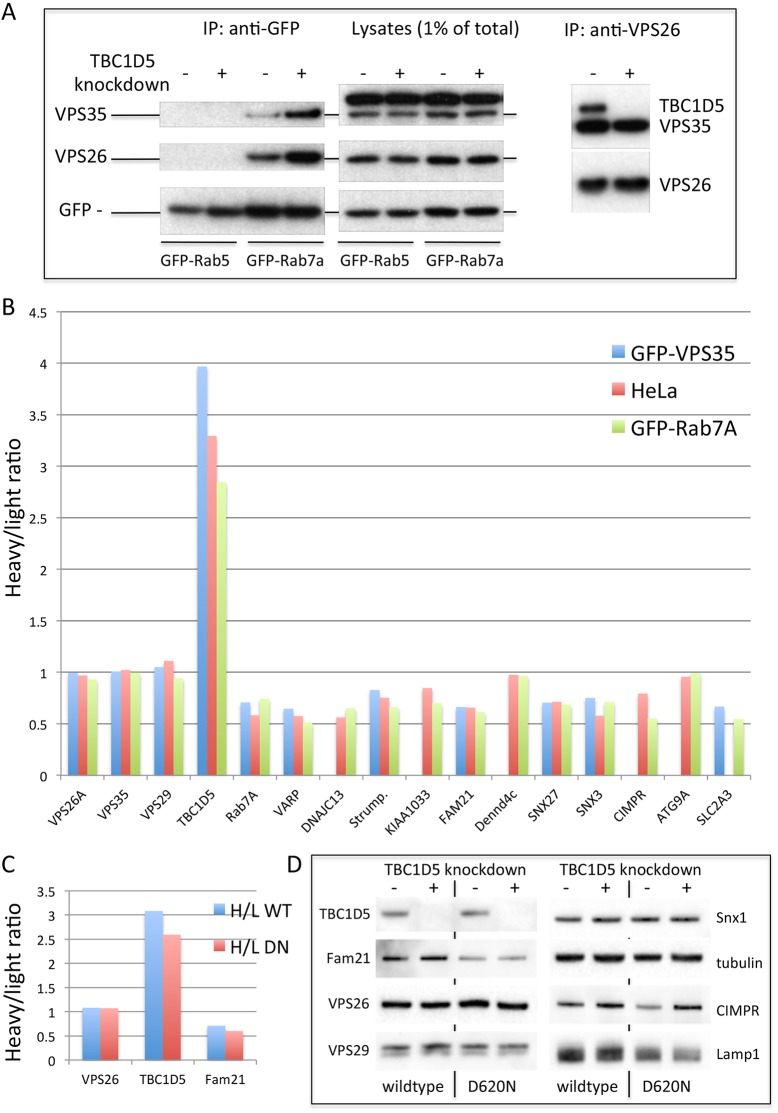
**TBC1D5 knockdown enhances the associations of the retromer CSC.** (A) Cells expressing either GFP-Rab5 or GFP-Rab7a were treated with siRNA to silence TBC1D5 expression. After lysis under native conditions, the GFP-tagged proteins were recovered by immunoprecipitation (IP) using anti-GFP. The immunoprecipitated proteins were analysed by western blotting. Retromer CSC proteins were detected in the GFP-Rab7a IP but not GFP-Rab5 IP. The knockdown of TBC1D5 increased the levels of retromer CSC proteins associated with GFP-Rab7a but no changes in protein levels were observed after TBC1D5 knockdown when lysates were analysed. IP of VPS26 confirmed that TBC1D5 expression was abolished and that loss of TBC1D5 does not alter the interaction between VPS26 and VPS35. (B) Following a protocol for SILAC labelling, three HeLa cell lines were labelled over several passages with heavy or light amino acids before being subjected to TBC1D5 knockdown (in the light amino acid-labelled cells) and treated with the DSP crosslinking reagent. After lysis, VPS26 was recovered by IP and the resulting IPs analysed by mass spectrometry. Levels of the proteins in the IPs are shown as a ratio of heavy:light normalised to VPS26. The retromer CSC proteins all give values close to 1, indicating that TBC1D5 knockdown does not affect retromer CSC assembly. The TBC1D5 protein has a ratio ∼3-3.5 consistent with a knockdown of the protein. Other proteins detected generally gave ratios <1, showing that more peptides labelled with light amino acids were detected, indicating an increased level of the protein after TBC1D5 knockdown. (C) Cells expressing either GFP-VPS35 WT or GFP-VPS35 D620N (DN) were treated as in B and the lysates were treated with anti-GFP antisera. The knockdown of TBC1D5 can enhance the interaction of the VPS35 D620N mutant with Fam21. (D) Lysates from cells in C analysed by western blotting to confirm TBC1D5 knockdown.

As Rab7a associates with the retromer CSC in a GTP-dependent manner (Harrison et al., 2014; Priya et al., 2015), we investigated whether increased Rab7a-GTP would enhance the association of Rab7a with the retromer CSC by silencing of TBC1D5. The results of a native immunoprecipitation of GFP-tagged Rab7a are shown in Fig. 3A. The retromer CSC proteins VPS35 and VPS26 co-immunoprecipitated with GFP-Rab7a, but not GFP-Rab5, and the levels of the retromer CSC proteins were increased in TBC1D5-knockdown cells. The elevated levels of retromer CSC on the endosomal membrane would be predicted to enhance the association of the retromer CSC with proteins that it is known to interact with on endosomes. Therefore, a protocol for stable isotope labelling with amino acids in cell culture (SILAC) was employed and cells were labelled with amino acids synthesised with either heavy or light isotopes of carbon and nitrogen over a number of passages to ensure that the amino acids were incorporated into the cellular proteins. TBC1D5 expression was silenced by RNAi in cells labelled with ‘light’ amino acids and, following treatment with a membrane-permeable chemical crosslinker, VPS26 was recovered from lysates by immunoprecipitation. The immunoprecipitates were analysed by mass spectrometry and the ratio of peptides detected that contained ‘heavy’ or ‘light’ amino acids was calculated. Fig. 3B shows the results obtained from three different cell lines. In each instance, the TBC1D5 heavy:light ratio is markedly above 1, indicating loss of protein from the light fraction consistent with the knockdown of TBC1D5. The ratios for the retromer CSC protein were all close to 1, which is to be expected. Most retromer-associated proteins, for example, those of the WASH complex (strumpellin, KIAA1033 and Fam21) generated ratios in the range of 0.7-0.5, indicating increased detection of those proteins in the TBC1D5-knockdown samples.

It has been shown that the Parkinson's disease (PD)-causing mutation in VPS35 (D620N) causes a reduction in association of the retromer CSC with the WASH complex protein Fam21 (Zavodszky et al., 2014; McGough et al., 2014). Therefore, we wondered if the elevated membrane association of the retromer CSC following TBC1D5 knockdown could enhance the interaction of the PD-causing VPS35 mutant with Fam21. Cells expressing either wild-type or D620N GFP-tagged VPS35 were labelled with heavy and light amino acids, treated with crosslinker and the lysates were immunoprecipitated with anti-GFP. In Fig. 3C, the graph shows that for VPS26, the heavy:light ratio is close to 1 as expected. Knockdown of TBC1D5 results in an increased ratio and, for Fam21, the ratio is decreased, indicating that the loss of TBC1D5 can enhance the association of both wild-type and D620N VPS35 with Fam21. A blot of lysates from TBC1D5-knockdown cells expressing GFP-VPS35 wild type or D620N is shown in Fig. 3D and indicates that enhanced association of Fam21 with both wild-type VPS35 and the D620N mutant is not caused by increased levels of Fam21 after TBC1D5 knockdown.

### Loss of TBC1D5 can rescue recruitment of the WASH complex in cells expressing PD-causing VPS35 D620N mutant

**Fig. 4.**
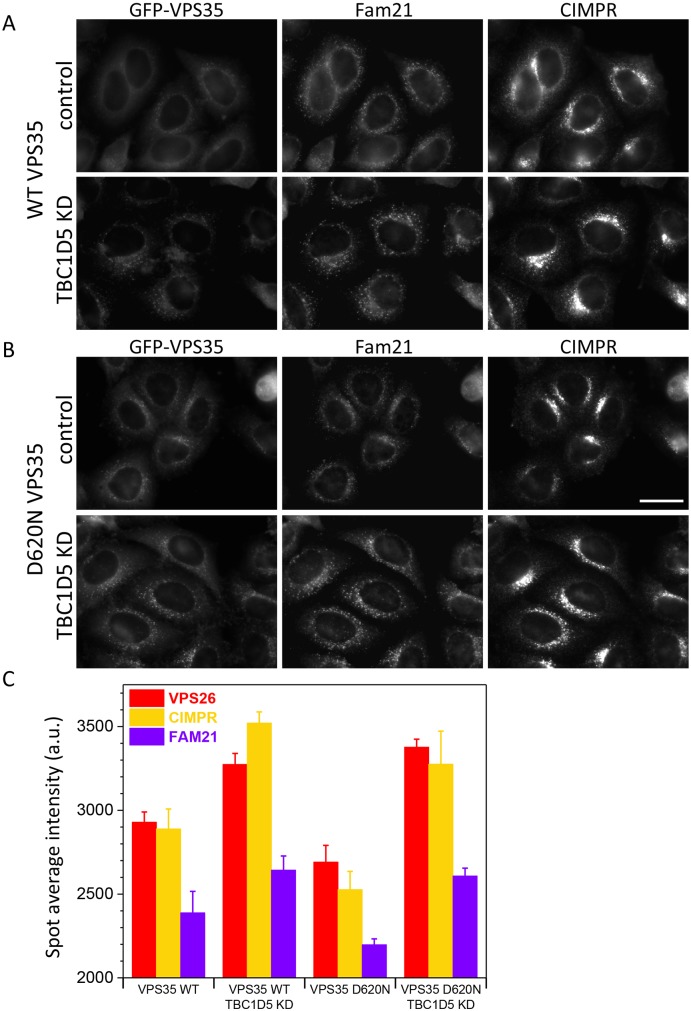
**Silencing TBC1D5 expression can rescue the impaired Fam21 interaction of the VPS35 D620N mutant.** (A,B) Cells expressing either wild-type GFP-VPS35 (WT) (A) or GFP-VPS35 D620N (DN) (B) were treated with siRNA to abolish TBC1D5 expression. After fixation, the cells were labelled with antibodies against Fam21 and the CIMPR. There is a marked increase in the fluorescence staining of Fam21 in the GFP-VPS35 D620N cells after TBC1D5 knockdown. Scale bar: 20 µm. (C) Cells from A and B were imaged using an automated microscope to measure the fluorescence intensity. Knockdown of TBC1D5 can rescue the fluorescence intensity of Fam21, consistent with an increase in the association of Fam21 (and the WASH complex) with the retromer CSC. *P*-values for TBC1D5 KD versus control are: VPS35 WT: VPS26, 2.7×10^−6^; CIMPR, 1.4×10^−6^; Fam21, 0.0022; VPS35 D620N: VPS26, 3.0×10^−8^; CIMPR, 9.8×10^−6^; Fam21, 8.5×10^−9^.

The retromer CSC functions as a hub to recruit proteins such as the WASH complex to endosomes (Harbour et al., 2010). The increased association of the retromer CSC with the WASH complex component Fam21 could enhance the recruitment of the WASH complex to endosomes. We have therefore investigated whether silencing TBC1D5 expression can increase recruitment of Fam21 to endosomes. Cells expressing either wild-type GFP-VPS35 (Fig. 4A) or the mutant D620N GFP-VPS35 (Fig. 4B) were treated with siRNA to silence TBC1D5 expression and then fixed and labelled with antibodies against Fam21 or the CIMPR. Fam21 staining was slightly reduced in the VPS35 D620N-expressing cells compared with the control cells expressing wild-type VPS35 (as expected) but was markedly brighter following knockdown of TBC1D5. Fig. 4C shows quantification of Fam21 staining following imaging using an automated microscope. As we have shown previously (see Fig. 1), knockdown of TBC1D5 enhances the fluorescence intensity of VPS26 and the CIMPR. Fam21 staining in the VPS35 D620N-expressing cells was markedly reduced compared with levels in cells expressing wild-type VPS35. Loss of TBC1D5 expression enhances the Fam21 fluorescence in both wild-type and D620N-expressing cells, but notably, the increase in Fam21 staining in the cells expressing the VPS35 D620N mutant results in levels of Fam21 staining that are almost the same as cells expressing wild-type VPS35. The gain in intensity for Fam21 staining was found to be statistically significant and the *P*-values are shown in the legend for Fig. 4C.

### Knockdown on TBC1D5 can lead to a gain of function for the retromer CSC

**Fig. 5.**
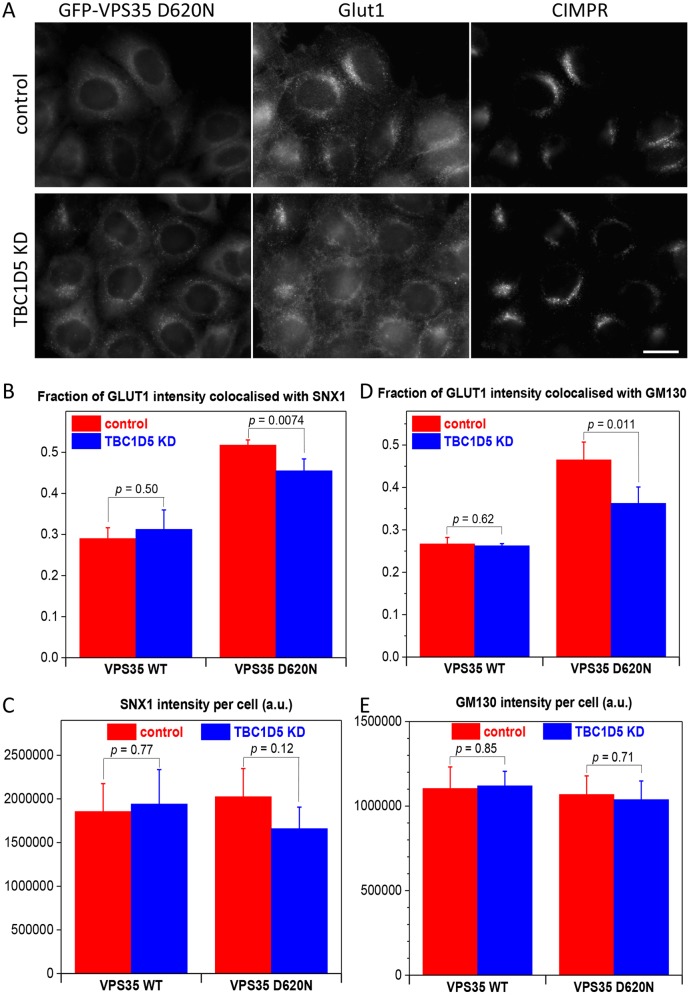
**Inhibition of TBC1D5 function can partially rescue the trafficking defects caused by the VPS35 D620N mutation.** (A) HeLa cells expressing GFP-VPS35 D620N were treated with siRNA to silence TBC1D5 expression. After fixation, the cells were labelled with antibodies against Glut1 and the CIMPR. The localisation of Glut1 appears to be shifted away from perinuclear structures after TBC1D5 knockdown. Scale bar: 20 µm. (B) HeLa cells expressing either GFP-VPS35 wild type (WT) or the GFP-VPS35 D620N mutant were treated with siRNA to silence TBC1D5 expression and then labelled with antibodies against Glut and Snx1. The cells were imaged using an automated microscope and the overlap of the Glut1 and Snx1 antibodies measured. There is more overlap of Glut1 with Snx1 in cells expressing VPS35 D620N. The overlap is reduced upon TBC1D5 knockdown but not to levels seen in cells expressing wild-type VPS35. (C) There is no appreciable change in the Snx1 fluorescence following TBC1D5 knockdown. (D) Cells treated as in B were labelled with anti-Glut1 and anti-GM130. (E) There is no appreciable change in GM130 fluorescence after TBC1D5 knockdown. For B-E, values are mean±s.d. and *P*-values are shown on the graphs.

Retromer function has been shown to be important for trafficking and localisation of a number of proteins including the CIMPR, sortilin, SorL1, Atg9a and Glut1 (Seaman, 2012; Zavodszky et al., 2014). In cells where retromer function is compromised, Glut1 accumulates in intracellular compartments that are positive for the SNX-BAR protein Snx1, or the lysosomal protein Lamp1 (Steinberg et al., 2013; Zavodszky et al., 2014). It has also been reported that loss of Fam21 function results in Glut1 being mistrafficked from endosomes to the Golgi (Lee et al., 2016). We report here that TBC1D5 knockdown can enhance the membrane association of the retromer CSC and can rescue the reduction in Fam21 recruitment to endosomes in cells expressing the VPS35 D620N mutant. Thus, we wondered whether TBC1D5 knockdown could rescue the trafficking of Glut1 in cells expressing the VPS35 D620N mutant. Cells expressing the VPS35 D620N mutant were treated with siRNA to silence TBC1D5 expression. In Fig. 5A, cells expressing the VPS35 D620N mutant were treated with siRNA to silence TBC1D5 expression. We observed that loss of TBC1D5 appears to reduce the extent of Glut1 localisation to the perinuclear compartment that was observed in control cells. To quantify this change on Glut1 localisation, cells expressing either wild-type GFP-VPS35 or the D620N mutant were treated with siRNA to abolish TBC1D5 expression. After fixation, the cells were stained with antibodies against Glut1 and either Snx1 or the Golgi marker protein GM130 and then imaged using an automated microscope. In Fig. 5B, the expression of the D620N mutant causes increased colocalisation of Glut1 with Snx1 relative to cells expressing wild-type VPS35. The fraction of Glut1 fluorescence that colocalises with Snx1 is reduced after loss of TBC1D5 expression. A similar result was obtained for Glut1 and GM130 (see Fig. 5D) and both sets of observations are statistically significant. The reduction in the fraction of Glut1 that colocalises with intracellular markers such as Snx1 or GM130 is consistent with increased localisation of Glut1 to the cell surface, although there does not appear to be a full rescue of the phenotype produced by the D620N mutant. In neither case can the reduced colocalisation of Glut1 with Snx1 or GM130 be due to altered levels of Snx1 or GM130, as any changes observed are not statistically significant (see Fig. 5C,E). We also observed that colocalisation of Glut1 with Lamp1 was reduced after TBC1D5 knockdown (see Fig. S1A), although for this experiment the cells were imaged using a conventional fluorescence microscope and the colocalisation was determined using the Volocity software package. Knockdown of TBC1D5 does appear to enhance Glut1 levels, although the increase was not statistically significant (Fig. S1B).

**Fig. 6.**
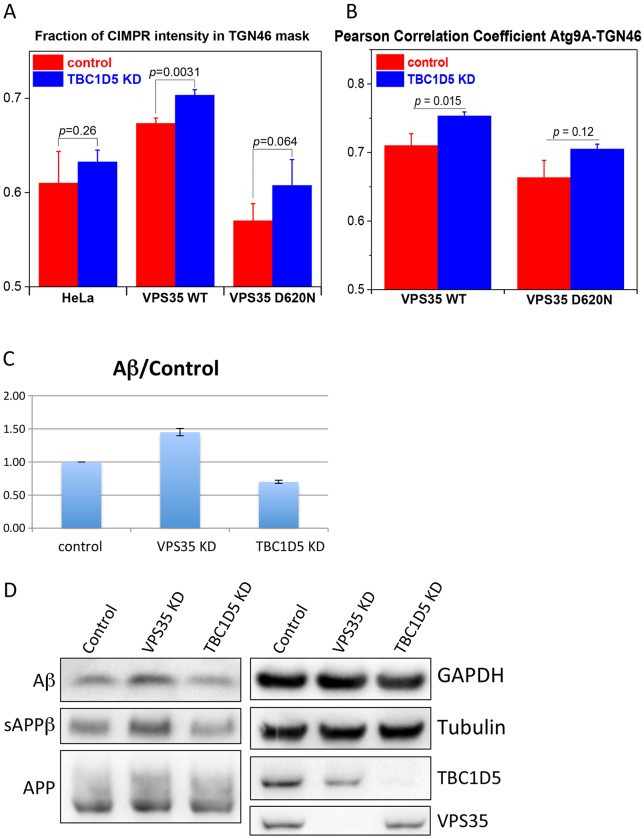
**Knockdown of TBC1D5 can enhance retromer function.** (A) Three different cell lines were treated with siRNA to knockdown TBC1D5 expression. Following fixation, cells were labelled with antibodies against CIMPR and TGN46 and then imaged using an automated microscope. The fraction of CIMPR present in a TGN46 mask is shown graphically. For each of the cell lines, knockdown on TBC1D5 enhances the colocalisation of CIMPR with TGN46 but only the GFP-VPS35 cells demonstrate statistical significance. Values are mean±s.d. and *P*-values for knockdown versus control are shown for each cell line. (B) TBC1D5 expression was silenced in cells expressing GFP-VPS35 wild type or the D620N mutant. Following fixation, the cells were labelled with antibodies against Atg9A and TGN46 and then imaged using an automated microscope. The Pearson correlation coefficient for Atg9A-TGN46 mask is shown graphically. For each of the cell lines, knockdown of TBC1D5 enhances the colocalisation of Atg9A with TGN46 but only the GFP-VPS35 wild-type cells demonstrate statistical significance. Values are mean±s.d. and *P*-values for knockdown versus control are shown for each cell line. (C) HEK293 cells stably expressing APPswedish were treated with siRNA to silence VPS35 or TBC1D5. Cell culture medium was collected and analysed for the Aβ peptide by western blotting. Knockdown of VPS35 increases APP processing to Aβ but loss of TBC1D5 expression has the opposite effect. The data shown are from two independent experiments that were highly reproducible. Values are mean±s.d. For both the VPS35- and TBC1D5-knockdown conditions, *P<*0.01 using Student's *t*-test compared with control. (D) Representative blots of media (for Aβ and sAPPβ) and lysates (for APP, VPS35, TBC1D5 and the loading controls, GAPDH and tubulin) from C showing the reduction in Aβ detected when TBC1D5 is silenced. There is also a reduction in sAPPβ.

The reduced colocalisation of Glut1 with Snx1, GM130 and Lamp1 after silencing TBC1D5 in the GFP-VPS35 D620N cells indicates that loss of TBC1D5 can enhance retromer function. We tested whether increased retromer function could enhance protein sorting at endosomes by using two methods. First, we determined whether TBC1D5 knockdown could enhance the colocalisation of retromer cargo proteins (e.g. CIMPR, Atg9a and CD8-SorL1) with the TGN marker protein TGN46. Fig. 6A shows that knockdown of TBC1D5 enhances the colocalisation of the CIMPR with TGN46 although the degree of enhancement is modest and not statistically significant in all the cell lines tested. The colocalisation of Atg9a, a protein that cycles through the endocytic system to the TGN, is also enhanced after TBC1D5 knockdown (see Fig. 6B), a gain that is not due to changes in levels of Atg9a (see Fig. S1D,E). It is worth noting, however, that increased Atg9a and TGN46 colocalisation is not statistically significant in cells expressing the VPS35 D620N mutant that causes impaired Atg9a trafficking (Zavodszky et al., 2014). Not all retromer cargo proteins respond to TBC1D5 knockdown equally and in cells expressing CD8-SorL1, we observed that loss of TBC1D5 expression very modestly reduced the colocalisation of the CD8-SorL1 reporter with TGN46 (see Fig. S2). Secondly, we tested whether loss of TBC1D5 expression could alter the processing of APP to Aβ. It has been shown previously that knockdown of VPS35 increases processing of APP to Aβ (Muhammad et al., 2008) and we find this also. In cells where TBC1D5 has been silenced, we observed a reduction in the levels of Aβ secreted into the medium and lower levels of sAPPβ, consistent with enhanced retromer function (Fig. 6C,D).

## DISCUSSION

In this study we tested the hypothesis that loss of TBC1D5 function will enhance the activity of the retromer complex by increasing the levels of membrane-associated retromer CSC proteins necessary for sorting cargo into tubules. We show that the loss of TBC1D5 expression upon RNA interference does indeed increase the levels of endosomally localised VPS26 and VPS35 proteins. As these proteins are components of the retromer CSC, which is a hub for recruiting other machinery to endosomes, we found that increasing the endosomal retromer CSC by TBC1D5 knockdown also enhances the association of the retromer CSC with accessory proteins such as Fam21 (and other components of the WASH complex, e.g. strumpellin and KIAA1033), Rme-8 (also known as DNAJC13), Snx3 and Snx27. In fact, loss of TBC1D5 expression can rescue the recruitment of Fam21 to the endosome in cells expressing the PD-causing VPS35 D620N mutant. The interaction of Fam21 with the retromer CSC requires the binding of VPS29 to VPS35, and TBC1D5 interacts with VPS29 (Helfer et al., 2013; Harbour et al., 2010). Thus, it is possible that the gain in association of the retromer CSC with Fam21 after loss of TBC1D5 expression could be due to a reduction in steric hindrance imposed by TBC1D5 binding to VPS29. However, we feel that this is unlikely as GFP-tagged versions of TBC1D5 and the Fam21 tail both coimmunoprecipitate not only retromer proteins but Fam21 (and other WASH complex proteins) and TBC1D5, respectively (Freeman et al., 2014). Additionally, proteomic analyses have revealed that expression levels of Fam21 and TBC1D5 are a fraction of that of retromer proteins (i.e. VPS35 or VPS29) (Itzhak et al., 2016) and thus loss of TBC1D5 expression will not markedly increase binding sites for Fam21 on the retromer CSC as the retromer CSC already outnumbers TBC1D5 and Fam21 by approximately 60:1 and 20:1, respectively (see Fig. S3).

Loss of TBC1D5 expression would be expected to lead to increased activation of Rab7a and this is what we observe. This, in turn, results in increased association of the retromer CSC (a Rab7a effector), with Rab7a and most likely accounts for the elevated levels of endosomally localised retromer CSC proteins. Increased active Rab7a would be predicted to also lead to changes in lysosome function and/or morphology as Rab7a is a key regulator of lysosomes (Basuray et al., 2012; Guerra and Bucci, 2016). We did observe changes in the morphology of lysosomes (using the protein Lamp1 as a marker) after TBC1D5 knockdown in line with changes elicited by knockdown of Rab7a itself (see Fig. S4). Studies in yeast have shown that the membrane-associated SNX-BAR dimer of Vps5p-Vps17p can displace yeast Rab7 (Ypt7p) from the retromer CSC and thereby coordinate the cargo selection activity of the retromer CSC with tubule formation (Purushothaman et al., 2017). Yeast do not have an obvious TBC1D5 homologue, however, and the yeast retromer complex is a much tighter association of the SNX-BAR dimer with the retromer CSC than has been observed in mammalian cells (Harbour and Seaman, 2011; Seaman et al., 1998). Therefore, it seems likely that the increased association of Rab7a with the retromer CSC in mammalian cells after loss of TBC1D5 will not dramatically alter the association of the retromer CSC with the SNX-BAR dimer in mammals as this is already a very transient association (Swarbrick et al., 2011).

Although TBC1D5 can modulate Rab7a activity, it is not the only reported GTPase-activating protein for Rab7a as both TBC1D15 and the Rac1 effector Armus have been reported to function as GAPs for Rab7a (Zhang et al., 2005; Frasa et al., 2010). Hence, it is possible that some of the potential negative consequences of the loss of TBC1D5 could be mitigated by compensatory effects mediated through TBC1D15 and Armus. While this article was in preparation, a study from Steinberg and colleagues reported that loss of TBC1D5 function results in a hyper-activated Rab7a (Jimenez-Orgaz et al., 2018), which demonstrates increased binding to RILP, a Rab7a effector. Our results broadly agree with theirs in that we observe an increase in activated Rab7a after loss of TBC1D5 expression along with enhanced association of Rab7a with an effector, namely the retromer CSC. Our data is also in agreement with the study from Steinberg and colleagues with respect to effects on the trafficking of retromer cargo proteins such as Glut1 but their study was focused on the effect of increased Rab7a activity on mitophagy – the autophagic clearance of mitochondria – where they showed that loss of TBC1D5 could enhance Rab7a-dependent mitophagy. Our study and that of Steinberg and colleagues do differ with respect to how loss of TBC1D5 affects the endosomal localisation of the retromer CSC as they do not observe an increase in endosomal VPS35 staining in TBC1D5 knockout (KO) cells. This difference could be due to their use of knockout cells versus our knockdown cells because a genetic KO can be compensated for through adaptations in the cells used for the KO. As KOs take days to generate along with weeks for generating clonal cell lines, there is scope for genetic adaptation to occur in a KO that would be less likely to occur in an RNAi-mediated knockdown over a period of 72 h. For example, expression of other GAPs such as TBC1D15 or Armus may increase to mitigate some of the effects of TBC1D5 KO, but may not do so in the context of a knockdown.

Previous studies have reported that induction of autophagy can reduce the association of TBC1D5 with the retromer CSC and that TBC1D5 has a role in autophagy (Popovic et al., 2012; Popovic and Dikic, 2014). Interestingly, it has been reported that induction of autophagy can lead to increased Glut1 trafficking to the cell surface (Roy and Debnath, 2017; Roy et al., 2017). We show that loss of TBC1D5 can reduce the amount of endosomally localised Glut1 and can enhance the colocalisation of the CIMPR with TGN46.

Interestingly, although levels of endosomally localised Fam21 are enhanced after loss of TBC1D5 expression, trafficking of the Glut1 protein to the cell surface, which is dependent upon Fam21 function, is not wholly rescued, possibly indicating that either the D620N mutation has effects on retromer function not confined to the loss of interaction with the WASH complex or that TBC1D5 function may be required for proper localisation of membrane proteins that pass through the endosome. There is some evidence to suggest that loss of TBC1D5 can cause mislocalisation of integrin proteins (Jia et al., 2016) but in our experiments we have not observed any pronounced deleterious effects on protein trafficking after loss of TBC1D5 function. When we investigated the localisation of the classical retromer cargo protein, the CIMPR, we found that there was a modest increase in the colocalisation of the CIMPR with the TGN marker protein, TGN46. It is not likely, however, that enhanced retromer function through TBC1D5 inhibition would markedly increase the levels of TGN-localised retromer cargo proteins as this would require increased production of transport intermediates and elevated docking and fusion of these intermediates with the TGN, and loss of TBC1D5 appears to enhance retromer function only at the endosome.

Another reason why the increase in colocalisation between the CIMPR and TGN46 is relatively modest is possibly due to the increase in CIMPR levels after TBC1D5 knockdown that is detectable both by increased CIMPR fluorescence staining and also by western blotting. This increase in CIMPR levels may actually result in elevated endosomal CIMPR due to saturation of the retrieval machinery (as would be observed if the CIMPR were simply overexpressed), thereby masking a more pronounced gain in TGN-localised CIMPR. Consistent with a gain of function for retromer after TBC1D5 knockdown is the observation that processing of APP to Aβ is reduced. This is, in some ways, very similar to the report that a pharmacological chaperone that stabilises the retromer CSC and enhances levels of membrane-associated VPS35 and VPS26 can also reduce APP processing to Aβ (Mecozzi et al., 2014) and confirms the importance of the retromer CSC in regulating the processing of APP.

Although somewhat speculative, it is tempting to suggest that the interaction between the retromer CSC and TBC1D5 could therefore be an attractive target for therapeutic intervention in neurodegenerative diseases such as Parkinson's disease and Alzheimer's disease. The apparent lack of marked trafficking defects when TBC1D5 is silenced, along with increased recruitment of retromer-associated proteins and reduced APP processing could provide an alternative avenue to explore for those seeking to modulate retromer function in disease states. Further work will be required, however, to develop an effective compound, although the recent structural studies of the TBC1D5-retromer CSC complex (Jia et al., 2016) could enable the identification of a small-molecule inhibitor that could target and disrupt the interaction, thereby mimicking the loss of TBC1D5 function that can be achieved using RNAi.

## MATERIALS AND METHODS

### Reagents and antibodies

Most general reagents used in this study were sourced from Sigma-Aldrich. The siRNA oligonucleotides were purchased from Dharmacon. Primary antibodies used in this study were as follows: anti-TBC1D5 [Santa Cruz, sc-376296, dilution 1:400 or 1:1000 for immunofluorescence (IF) microscopy or western blotting (WB), respectively], anti-VPS26 (Abcam, ab23892, 1:800 IF or 1:1000 WB), anti-VPS35 [Santa Cruz, sc-374372, 1:800 IF or 1:1000 WB, or from the Seaman lab (see Seaman, 2007), 1:300 for IF], anti-CIMPR (Abcam, ab2733, 1:400 IF or 1:1000 WB), anti-Lamp1 (Santa Cruz, sc-18821, 1:500 IF or 1:1000 WB), anti-Glut1 (Abcam, ab15309, 1:400 IF), anti-GM130 (BD Transduction labs 610822, 1:500 IF), anti-Fam21 (Millipore, ABT79, 1:400 IF or 1:1000 WB), anti-Aβ (Covance, SIG-39320, 1:1000 WB), anti-sAPPβ (IBL America, 10321, 1:800 WB), anti-Rab7a:GTP (NewEast Biosciences, 26923, 1:300 IF), anti-TGN46 (Seaman lab, see Seaman, 2007, dilution 1:600 IF), anti-GFP (Seaman lab, see Seaman et al., 2009, 1:1000 for immunoprecipitation), anti-Snx1 (BD Transduction labs, dilution 1:400 IF or 1:1000 WB) and anti-Tubulin (Sigma-Aldrich, dilution 1:1000 WB). Secondary fluorescently labelled antibodies were purchased from Invitrogen.

### Cell lines and cell culture

The HeLa cells used in the study are a variant called HeLaM (Tiwari et al., 1987) and have been used previously in studies from the Seaman lab. Cells stably expressing GFP-Rab7a wild-type, Q66L and T22N, GFP-Rab5 or GFP-Rab9 have been described previously (Seaman et al., 2009). Cells stably expressing GFP-Snx3 have been described previously (Vardarajan et al., 2012) and cells stably expressing GFP-VPS35 wild-type or D620N have been described in Zavodszky et al. (2014). The HEK293 cells stably expressing APPswedish were generously provided by Professor Peter St-George-Hyslop (Cambridge Institute for Medical Research, University of Cambridge). Cells were maintained in DMEM supplemented with 10% fetal calf serum, glutamine containing penicillin and streptomycin. Stably transfected cells were maintained as above but G418 was added to the medium to a final concentration of 0.5 mg/ml.

For the SILAC-based experiments, cells were cultured in SILAC medium that lacked leucine, lysine and arginine. Amino acids synthesised with either heavy or light isotopes of carbon and nitrogen were added along with dialysed fetal calf serum. The cells were maintained in the SILAC medium and passaged at least four times before being used in the respective experiments.

### Immunofluorescence, quantitative imaging and automated microscopy

For conventional immunofluorescence (e.g. Fig. 1A), cells were seeded onto coverslips 24 h prior to fixation using 4% paraformaldehyde in PBS and permeabilisation with 0.1% Triton X-100 in PBS. After labelling with primary and secondary antibodies (diluted in PBS with 3% BSA), the coverslips were mounted onto glass slides with ProLong mounting medium (Invitrogen). Cells were imaged using a Zeiss AxioPlan microscope with a ×63 PlanAPO objective lens. Images were captured through a Hamamatsu CCD camera controlled via the manufacturer's software.

For automated microscopy, control or siRNA-silenced cells were seeded in 24-well plates (CELLSTAR^®^, Greiner-Bio, Stonehouse, UK) before fixation 24 or 48 h post-seeding at 50-70% cell confluency. Cells were permeabilised, unspecific antibody binding was blocked and cells were stained as for regular immunofluorescence. After the secondary antibody staining, cells were washed and then stained with a whole cell stain (Whole Cell Stain Blue, Thermo Fisher) diluted 1:500 in PBS from a DMSO stock solution for 30 min at room temperature. Finally, cells were washed twice with PBS and overlaid with 1 ml fresh PBS per well before imaging or storage at 4°C until imaging.

Cells were imaged on a Thermo Fisher high-content imaging platform, either a Cellomics Arrayscan Vti or a Cell Insight CX7, using a 40× or a 20×0.6 NA objective. Data were acquired from 250 cells per well, with the smoothened and intensity-thresholded whole cell stain image used to define the cells, using the spot detector or colocalisation bio-application in the Thermo HSC Studio software and sequential acquisition of the three- or four-colour images with multi-line filters. Relevant field average parameters were exported and analysed in Origin software (OriginLab Corporation, Northampton, MA, USA). For most experiments presented, biological replicates were analysed in multiple (usually 4) wells, and the statistics shown represent one-way ANOVA analysis for the average values obtained from these replicates and calculated in Origin software. For the Rab7-GTP staining, a single well with 250 cells was analysed for each cell line and condition (control or TBC1D5 KD). For the Glut1-Lamp1 colocalisation analysis images acquired on a Zeiss inverted microscope were analysed in Volocity software (PerkinElmer, Waltham, MA, USA). Images were intensity-thresholded before colocalisation analysis.

### Transfections

Transient transfections of the HeLa cells were performed using polyethylenimine (PEI) as described in Harbour et al. (2010). The siRNA knockdowns were performed using siRNA oligos purchased from Dharmacon following a protocol also described in Zavodszky et al. (2014).

### Crosslinking, immunoprecipitation and western blotting

Cells in 140 mm dishes were washed with PBS twice before the addition of PBS containing 1 mM CaCl_2_ and 1 mM magnesium acetate. The crosslinking reagent DSP [dithiobis(succinimidyl propionate)] (ThermoFisher) dissolved in DMSO was then added to the cells to a final concentration of 0.5 mM and crosslinking allowed to proceed for 20 min at room temperature. The cells were then washed twice with PBS containing 5 mM Tris-HCl before lysis. For immunoprecipitation experiments, cells were harvested from 140 mm tissue culture dishes using a cut rubber bung to scrape the cells into lysis buffer (20 mM Hepes-KOH, pH 7.2, 50 mM potassium acetate, 200 mM sorbitol and 2 mM EDTA with 0.1% Triton X-100 for the native immunoprecipitation and Tris-buffered saline with 0.5% Triton X-100 for the non-native immunoprecipitation following crosslinking). The lysate was centrifuged at 10,000 ***g*** for 5 min to pellet insoluble material and then incubated with Protein-A Sepharose for 30 min as a preclearing step. Following a second spin at 10,000 ***g*** for 5 min, the lysate was then treated with antibodies against the target proteins for 90 min after which Protein-A Sepharose was added to capture the immune complexes. The Protein-A Sepharose was subjected to multiple washes before desiccation in a speed vac and then analysis by western blotting or by mass spectrometry.

Western blotting with [^125^I]-Protein-A detection: samples were subjected to SDS-PAGE and then immobilised on nitrocellulose by electrophoretic transfer. After washes with a TBS-based blocking buffer containing gelatin, the nitrocellulose was cut into strips and then incubated with primary antibodies. After washes, the [^125^I]-Protein-A was added to a final dilution of 1:1000. Following incubation on a rocking platform, the strips were again washed and then arranged for exposure to X-ray film.

Western blotting by ECL detection: cell lysate proteins were separated by electrophoresis on a 4-12% Bis-Tris gel (Nupage, Invitrogen) in MES/SDS buffer at 150 V for 70 min before transfer onto nitrocellulose membranes (GE Healthcare). Non-specific antibody binding was blocked by incubating the membranes for 1 h in TBS with 0.1% Tween20 (TBST) containing 5% skimmed milk powder. Membrane strips were then incubated with primary antibodies diluted in TBST/milk for 1-2 h at room temperature. After three washes with TBST, membranes were incubated with HRP-conjugated secondary antibodies (Sigma) in TBST/milk for 1 h. After a further three washes with TBST, membranes were treated for 1 min with peroxide and Luminol reagents (Millipore, Billerica, MA) before imaging using a Bio-Rad ChemiDoc Imager.

### Mass spectrometry

Detailed information pertaining to the mass spectrometry can be found in Tyanova et al. (2014). Briefly, samples from the crosslinked immunoprecipitations were resolved ∼2 cm into a pre-cast SDS-polyacrylamide gel, the entire lane excised and cut into six equal slices. Proteins were reduced and alkylated then digested in-gel using trypsin. The resulting peptides were analysed by LC-MSMS using a Q Exactive (Thermo Scientific) coupled to an RSLC3000nano UPLC (Thermo Scientific) with the data acquired in a data-dependent acquisition (DDA) fashion. Raw files were processed in Maxquant 1.5.2.8 using the default setting for a SILAC duplex experiment with re-quantify enabled.

## Supplementary Material

Supplementary information
