# Supplementary material for: Inhibition of TBC1D5 activates Rab7a and can enhance the function of the retromer cargo-selective complex
Source: J Cell Sci. 2018 Jun 21;131(12):jcs217398. doi: 10.1242/jcs.217398 (PMC6031384; doi:10.1242/jcs.217398)
Supplement: Supplementary information [file joces-131-217398-s1.pdf]

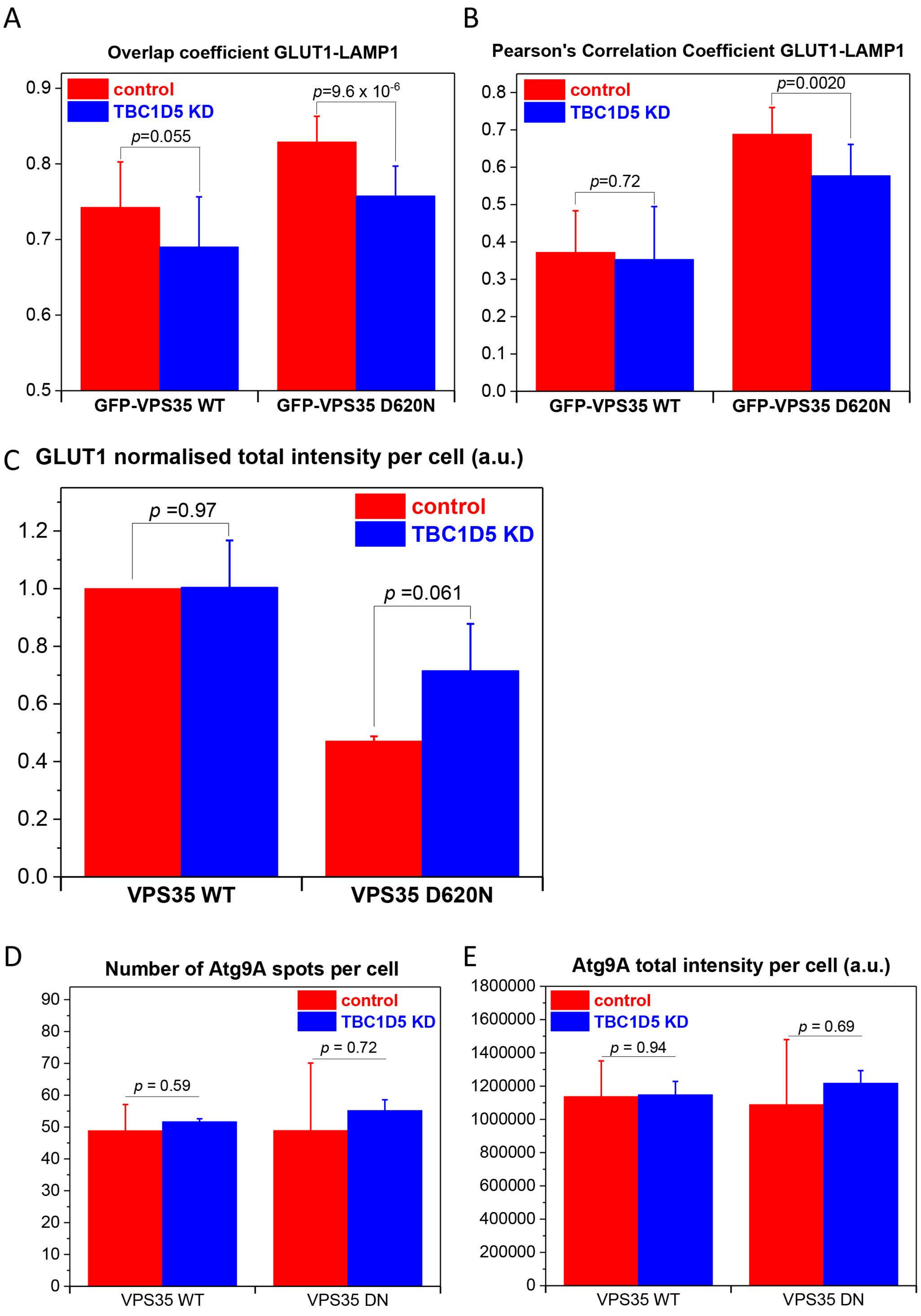
A
Overlap coefficient GLUT1-LAMP1
control
TBC1D5 KD
p=0.055
p=9.6 x 10-6
0.9
0.8
0.7
0.6
0.5
GFP-VPS35 WT
GFP-VPS35 D620N
B
Pearson's Correlation Coefficient GLUT1-LAMP1
control
TBC1D5 KD
p=0.72
p=0.0020
0.8
0.7
0.6
0.5
0.4
0.3
0.2
0.1
0.0
GFP-VPS35 WT
GFP-VPS35 D620N
C
GLUT1 normalised total intensity per cell (a.u.)
control
TBC1D5 KD
p =0.97
p =0.061
1.2
1.0
0.8
0.6
0.4
0.2
0.0
VPS35 WT
VPS35 D620N
D
Number of Atg9A spots per cell
control
TBC1D5 KD
p = 0.59
p = 0.72
90
80
70
60
50
40
30
20
10
0
VPS35 WT
VPS35 DN
E
Atg9A total intensity per cell (a.u.)
control
TBC1D5 KD
p = 0.94
p = 0.69
1800000
1600000
1400000
1200000
1000000
800000
600000
400000
200000
0
VPS35 WT
VPS35 DN

Figure S1. **A**. TBC1D5 was silenced by RNAi in cells expressing wildtype (WT) GFP-VPS35 or the D620N VPS35 mutant (tagged with GFP). Following fixation, cells were labelled with antibodies to Glut1 and Lamp1 and then imaged. Images were analysed using Volocity software to determine the overlap coefficient and the Pearson's Correlation Coefficient (in **B**) for Glut1 and Lamp1. Levels of Glut1 fluorescence were also determined and are shown in **C**. TBC1D5 was silenced by RNAi in cells expressing wildtype (WT) VPS35 or the D620N VPS35 mutant (DN), both tagged with GFP Following fixation, cells were labelled with antibodies to Atg9a and TGN46 and then imaged. The number of Atg9a spots per cell and the Atg9a total intensity was determined using automated microscopy and is shown respectively in **D** and **E**.

Journal of Cell Science • Supplementary information

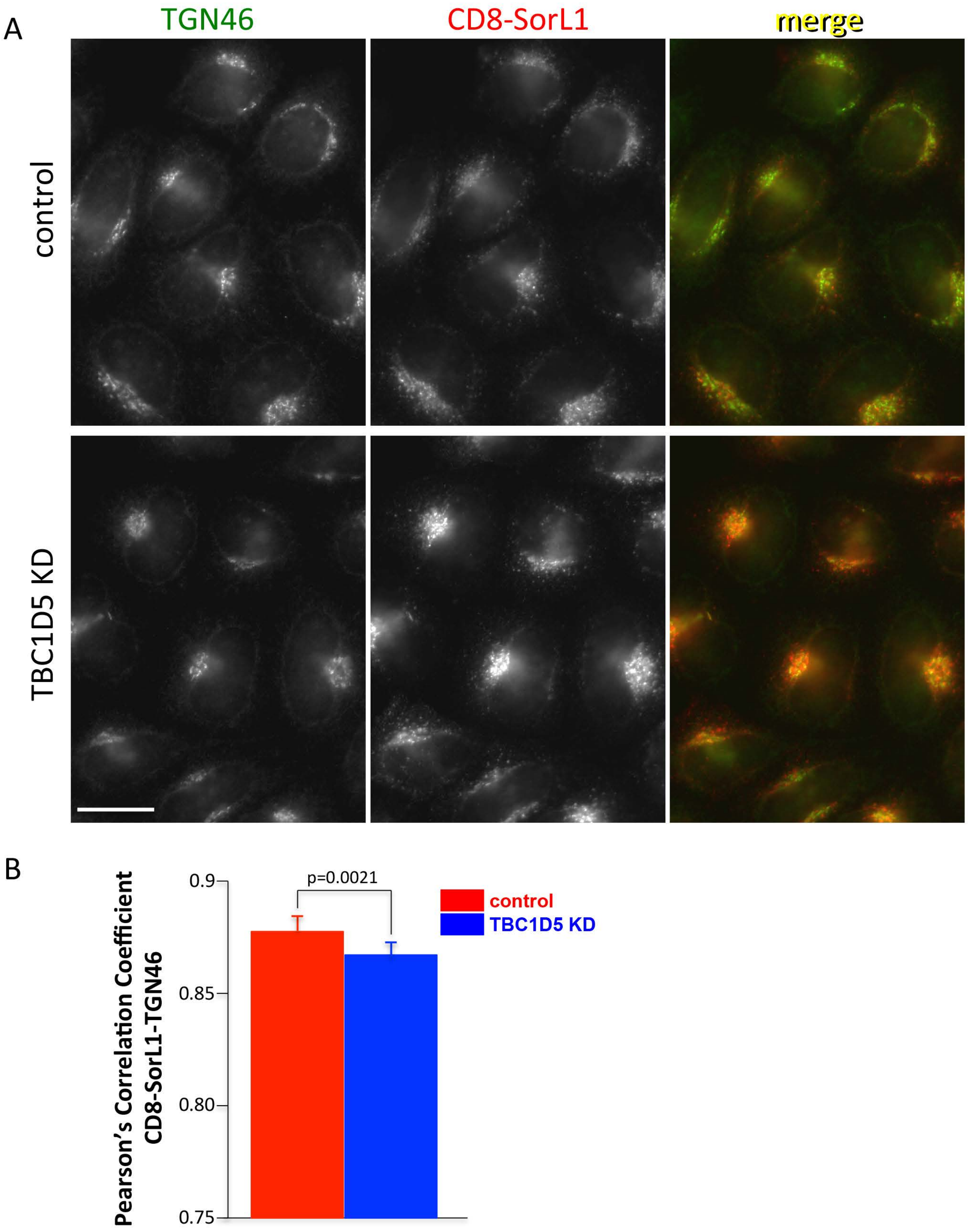

Figure S2. **A**. Cells expressing CD8-SorL1 were treated with siRNA to silence TBC1D5 expression. Following fixation, the cells were labelled with antibodies against CD8 and TGN46. The images shown are representative cells. Scale bar = 20 μm. **B**. Cells treated as in A were imaged using an automated microscope and the colocalisation between the CD8-SorL1 reporter and TGN46 determined using Pearson's Correlation Coefficient. Error bars are SD. Loss of TBC1D5 only slightly reduces the colocalisation of CD8-SorL1 with TGN46.

A

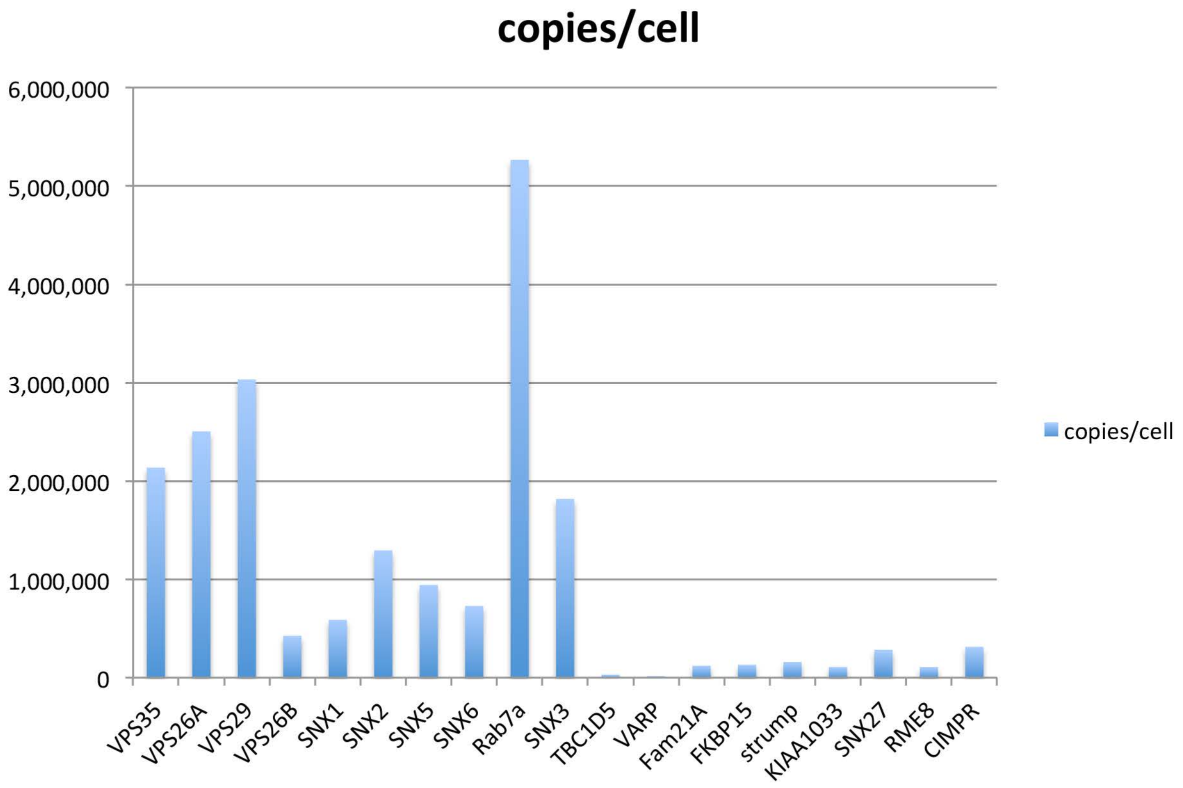

B

protein	copies/cell
VPS35	2,135,818
VPS26A	2,503,520
VPS29	3,032,390
VPS26B	426,044
SNX1	587,580
SNX2	1,294,191
SNX5	942,412
SNX6	728,590
Rab7a	5,267,880
SNX3	1,816,578
TBC1D5	30,032
VARP	15,984
Fam21A	121,104
FKBP15	130,545
strump	158,197
KIAA1033	107,917
SNX27	282,681
RME8	106,611
CIMPR	312,736

Figure S3. **A**. Graphical representation of the relative levels of retromer CSC and associated proteins using data from Itzhak et al., (2016). The TBC1D5 protein is expressed at relatively low levels compared to retromer CSC proteins. **B**. The actual numbers used to produce the graph shown in A.

Journal of Cell Science • Supplementary information

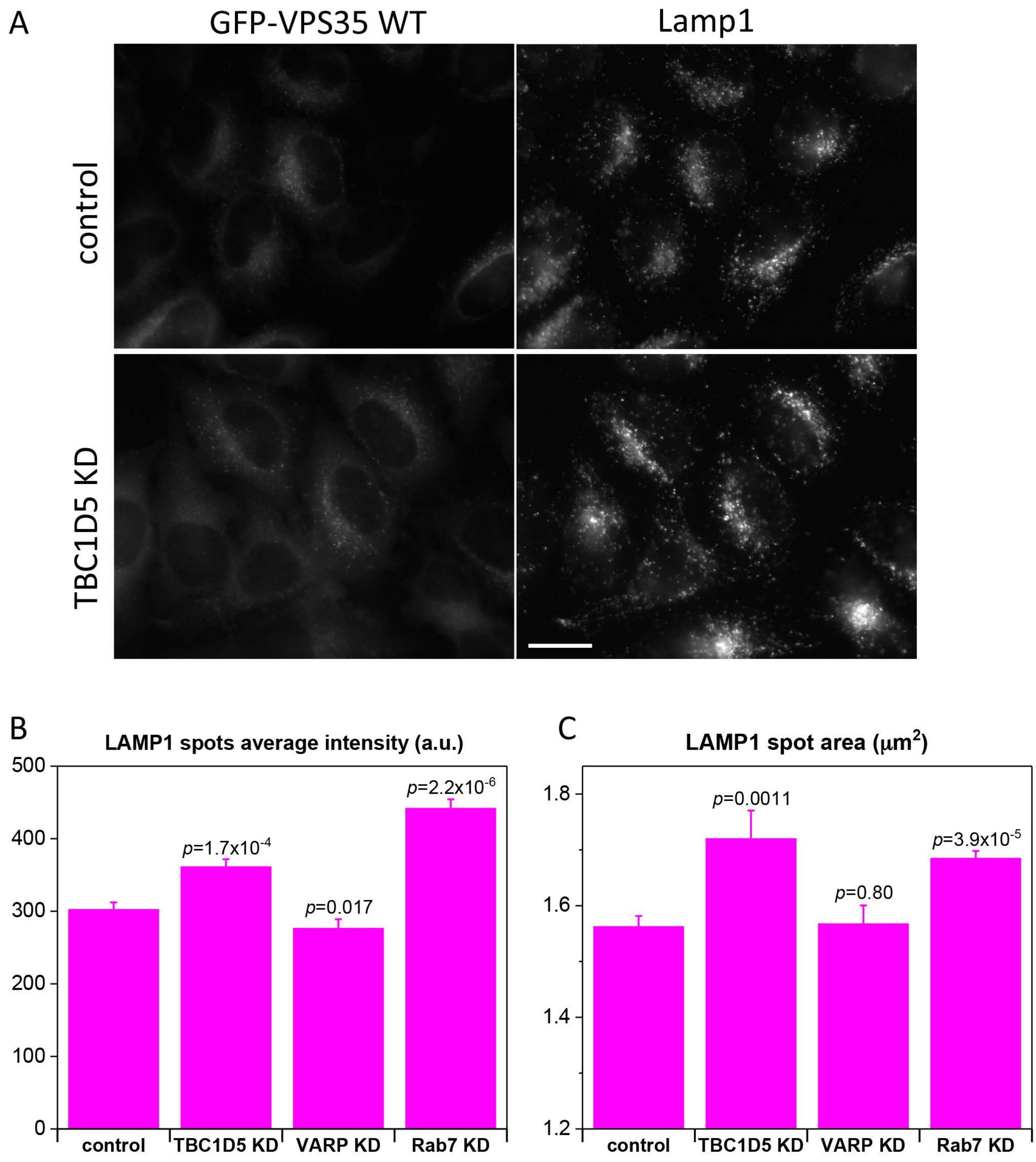

Figure S4. Loss of TBC1D5 expression affects lysosomal morphology. **A.** Cells expressing wildtype (WT) VPS35 (tagged with GFP) were treated with siRNA to silence TBC1D5 expression. After fixation the cells were labelled with antibodies against Lamp1. Representative images are shown indicating that Lamp1 fluorescence is brighter after TBC1D5 knockdown. Scale bar = 20 μm. **B** and **C**. Cells were treated with siRNA to silence expression of TBC1D5, VARP or Rab7a and then imaged using an automated microscope. Loss of either TBC1D5 or Rab7a both result in brighter Lamp1 staining (in B) and larger Lamp1-positive structures (in C). Error bars are SD, statistical significance is shown above each bar and compares to control.
